# More on the Tuskegee Study of Untreated Syphilis

**DOI:** 10.1200/GO.20.00340

**Published:** 2020-08-14

**Authors:** Robert M. White

**Affiliations:** Robert M. White, MD, Silver Spring, MD

## TO THE EDITOR:

I read with interest the recent article in *JCO Global Oncology* by Salhia and Olaiya.^1^ However, I have specific concerns that require historical corrections and clarifications about the Tuskegee Study of Untreated Syphilis (TSUS). The key points include: (1) the entities responsible for the TSUS; (2) the issue of denial of penicillin; (3) the term bad blood as an appropriate, culturally sensitive tool; and (4) participants’ knowledge about being studied. An item of interest will also be included to suggest the relevance of the TSUS to oncology.

First, the TSUS was a cooperative study initiated by the US Public Health Service (USPHS) and supported by Alabama State and Macon County public health authorities. Tuskegee Institute, the John A. Andrew Memorial Hospital, and the Tuskegee Veterans Hospital—all black-run institutions—coordinated local research resources and activities. The Milbank Memorial Fund, a New York City philanthropic organization, supplied the funding for autopsies and burial expenses; Tuskegee Institute disbursed the funds.^2,3^

Second, a review of one of the authors’ references reveals two tables listing TSUS patients who received treatment, including penicillin.^4^ An earlier TSUS article has a table including other patients who received arsenicals and adequate penicillin.^5^ It is unknown what information was given to the TSUS participants when they received these treatments. However, the information and/or the treatment experience seemed to be sufficient for subsequent TSUS investigators to decipher the participants’ histories and record the treatments in the tables.

Third, as documented in syphilology and physical diagnosis textbooks, medical and public health practice and educational materials, academic articles, and other sources, bad blood was syphilis. And, most important, syphilis was bad blood as indicated in routine doctor–patient interactions and health educational activities throughout the 1932 to 1972 duration of the TSUS.^6^ Accordingly, in physical diagnosis, the term bad blood was another equivalent for syphilis and in syphilology, “The patient should be informed of the diagnosis, and in doing so one must, depending upon the patient’s educational level, use such terms as can be understood as ‘syphilis,’ ‘the pox,’ ‘syph,’ and ‘bad blood.’”^7(p183)^ These and other appropriate uses of bad blood as a term for syphilis were not just definitive, but were in use in patient care, research, and venereal disease control activities across the United States.

Fourth, because of the ensuing outrage associated with the 1972 media exposure and the recruitment of the TSUS men into a future lawsuit,^8^ the public may not have had a reliable source to learn what the men were told about their 40 years of participation in the study. However, in 1958, the TSUS men received a 25-year anniversary certificate from the USPHS with the inscription, “In grateful recognition of 25 years of active participation in the *Tuskegee medical research study*” (*italics* added).^9^ This documented—in writing—what the men were told about their participation in the study.

John R. Heller, Jr, MD, recruited the presumably nonsyphilitic males into the TSUS, coauthored the first TSUS article in the 1936 volume of *Journal of the American Medical Association*, and rose to chief of the USPHS’ Venereal Disease Division. As chief of the Venereal Disease Division, Dr. Heller managed the development of penicillin, its testing, and its use in rapid treatment centers across the United States. After near eradication of syphilis, Dr. Heller became the Director of the National Cancer Institute—a transition from public service in syphilis, a curable disease, to a new challenge in cancer. In 1959, he was featured on the cover of *Time* magazine. In an article about emerging basic and clinical oncology research, Dr. Heller explained that in the cases of both penicillin and rapid treatment centers, “I worked myself out of a job.”^10^

The information herein may be useful in explaining what happened and did not happen in the TSUS. Correct information is important because citing the TSUS in the United States often results in: (1) general discussions of health disparities^11^; and (2) a health crisis when unequal outcomes develop unfavorably in black Americans, as in the current COVID-19 pandemic.^12^ Race-related disparities, which occur in prevalence, morbidity, and/or mortality rates, can result in the TSUS being cited (as a reason or as part of a historical pattern) by professionals or the general public. Accurate and complete historical information may mitigate the mistrust of health professionals and health care. This may move disadvantaged communities toward solutions for their disparate health conditions. Unfortunately, to do otherwise exposes these communities to the risk of becoming the so-called new TSUS victims in the COVID-19 pandemic. Here, a perceived withholding of treatment may occur as the result of their own, uninformed inaction.
